# Real-Time Algorithm for Detrended Cross-Correlation Analysis of Long-Range Coupled Processes

**DOI:** 10.3389/fphys.2022.817268

**Published:** 2022-03-11

**Authors:** Zalan Kaposzta, Akos Czoch, Orestis Stylianou, Keumbi Kim, Peter Mukli, Andras Eke, Frigyes Samuel Racz

**Affiliations:** ^1^Department of Physiology, Faculty of Medicine, Semmelweis University, Budapest, Hungary; ^2^Institute of Translational Medicine, Semmelweis University, Budapest, Hungary; ^3^Oklahoma Center for Geroscience and Healthy Brain Aging, Department of Biochemistry and Molecular Biology, University of Oklahoma Health Sciences Center, Oklahoma City, OK, United States; ^4^Department of Radiology and Biomedical Imaging, Yale University School of Medicine, New Haven, CT, United States; ^5^Department of Neurology, Dell Medical School, University of Texas at Austin, Austin, TX, United States

**Keywords:** detrended cross-correlation analysis, detrended fluctuation analysis, real-time, bivariate, fractal, long-range coupling, fractal connectivity

## Abstract

Assessing power-law cross-correlations between a pair – or among a set – of processes is of great significance in diverse fields of analyses ranging from neuroscience to financial markets. In most cases such analyses are computationally expensive and thus carried out offline once the entire signal is obtained. However, many applications – such as mental state monitoring or financial forecasting – call for fast algorithms capable of estimating scale-free coupling in real time. Detrended cross-correlation analysis (DCCA), a generalization of the detrended fluctuation analysis (DFA) to the bivariate domain, has been introduced as a method designed to quantify power-law cross-correlations between a pair of non-stationary signals. Later, in analogy with the Pearson cross-correlation coefficient, DCCA was adapted to the detrended cross-correlation coefficient (DCCC), however as of now no online algorithms were provided for either of these analysis techniques. Here we introduce a new formula for obtaining the scaling functions in real time for DCCA. Moreover, the formula can be generalized via matrix notation to obtain the scaling relationship between not only a pair of signals, but also all possible pairs among a set of signals at the same time. This includes parallel estimation of the DFA scaling function of each individual process as well, thus allowing also for real-time acquisition of DCCC. The proposed algorithm matches its offline variants in precision, while being substantially more efficient in terms of execution time. We demonstrate that the method can be utilized for mental state monitoring on multi-channel electroencephalographic recordings obtained in eyes-closed and eyes-open resting conditions.

## Introduction

Fractal dynamics are widely present both in man-made systems such as financial markets (He and Chen, 2011) or the flow of traffic (Zebende et al., 2011), as well as in natural phenomena including seismic activity (Shadkhoo and Jafari, 2009) or physiological processes including spontaneous neural fluctuations (Racz et al., 2021) or the variability of heart rate (Ivanov et al., 1996). Moreover, many processes express long-range correlations not only on the univariate level of their individual dynamics, but also in their bivariate coupling, such as the absolute values of returns of the Dow Jones and S&P500 indices (Podobnik et al., 2007), the fluctuations in oxygenated and deoxygenated hemoglobin compartments of cerebral blood volume (Mukli et al., 2018), or electrophysiological activities recorded simultaneously from various cortical regions (Stylianou et al., 2020). Assessing fractal coupling in these systems is often crucial for their better understanding as it can reveal characteristics and inner structures that otherwise would remain undetected by scale-dependent approaches (He and Chen, 2011; Zilber et al., 2012; Stylianou et al., 2020).

Given that assessment of long-range couplings can be of relevance for a broad range of scientific disciplines, many different methods and analysis techniques have been developed recently for this purpose. The first of such methods was introduced by Podobnik and Stanley (2008), termed detrended cross-correlation analysis (DCCA). DCCA was directly derived from detrended fluctuation analysis (DFA) of a single signal (Peng et al., 1994; Kristoufek, 2014a), for estimating the detrended covariance between two signals as the scale-invariant measure. Analogously to DFA, in case of long-range coupling between two signals the detrended covariance would also show power-law scaling with a bivariate scaling exponent (Podobnik and Stanley, 2008; Podobnik et al., 2009). Zhou (2008) shortly generalized DCCA to the multifractal domain along the lines of multifractal DFA (Kantelhardt et al., 2002). DCCA was extended further into a different direction: Zebende (2011) proposed to calculate the detrended cross-correlation coefficient (DCCC) as a measure superior to the Pearson-correlation coefficient for non-stationary series. Several alternative approaches have been proposed as well, such as the detrended moving average cross-correlation analysis (He and Chen, 2011) or the height cross-correlation analysis (Kristoufek, 2011), while it has also been shown that DCCC acts as a true cross-correlation coefficient, namely it is bounded between [−1, 1] and appropriate confidence intervals can be generated to assess its significance (Podobnik et al., 2011). Nevertheless, straightforward implementations of these algorithms are computationally expensive, which reduces their applicability in areas where real-time processing is key, such as in the case of financial markets (Demirer et al., 2020) or online mental state monitoring (Gateau et al., 2015). Despite the need for such tools, to the best of our knowledge no online algorithms have been proposed yet for the real-time computation of fractal cross-correlation coefficients.

The most computationally demanding step of obtaining long-range correlations is the assessment of the scaling function. Here we introduce a formula that allows for obtaining the bivariate scaling function in real-time, which constitutes the backbone of both univariate DFA, DCCA, and DCCC analysis. Furthermore, by generalizing the formula using matrix notation we open the method up for efficiently obtaining the scaling functions of all possible pairs in a set of simultaneously recorded signals. Our algorithm allows faster execution time with decreased memory usage while maintaining the precision of the previously mentioned offline solutions. We demonstrate our method’s use in mental state monitoring via the analysis of multi-channel electroencephalographic recordings obtained in eyes-closed and eyes-open resting conditions.

## Materials and Methods

Here we present a generalization of the real-time algorithm proposed by Hartmann et al. (2013) – which was introduced for performing online DFA analysis – to the bivariate domain. We show that our algorithm is equivalent with simple univariate DFA analysis when the bivariate scaling function of a time series is calculated with itself. Given that one can obtain both the uni- and bivariate scaling functions of two processes at the same time, the DCCC can thus also be obtained with ease.

### Offline Detrended Cross-Correlation Analysis

Let us consider two long-range cross-correlated time series *x*(*t*) and *y*(*t*), each of length *N*. First, both time series are integrated to obtain $X\left(t\right)=\sum_{i=1}^{t}x\left(i\right)$ and $Y\left(t\right)=\sum_{i=1}^{t}y\left(i\right)$. Then, the integrated time series are divided into *k* = *N* − *s* + 1 overlapping windows, each of length *s*, starting at *t* = *k* and ending at *t* = *k* + *s* − 1. The local trends ${\tilde{X}}_{j}$ and ${\tilde{Y}}_{j}$ are estimated in all windows *j* by ordinary least squares estimation. For example, local trend in the first window of *X*(*t*) has the form


(1) $${\tilde{X}}_{1}\left(t\right)=m\times t+b,$$

where the coefficients can be expressed as


$$m=\frac{n\sum_{i=1}^{n}X\left(i\right)i-\sum_{i=1}^{n}X\left(i\right)\sum_{i=1}^{n}i}{n\sum_{i=1}^{n}i^{2}-{\left(\sum_{i=1}^{n}i\right)}^{2}}$$

(2) $$b=\frac{\sum_{i=1}^{n}X\left(i\right)-m\sum_{i}^{n}i}{n}$$

according to Hartmann et al. (2013). The local trends are then utilized in calculating the covariance of the residuals in each box such as


(3) $$f_{DCCA}^{2}\left(s,j\right)=\frac{1}{s-1}\sum_{i=j}^{j+s-1}\left(X\left(i\right)-{\tilde{X}}_{j}\left(i\right)\right)\left(Y\left(i\right)-{\tilde{Y}}_{j}\left(i\right)\right).$$

Finally, the detrended covariance at scale *s* is defined (Podobnik and Stanley, 2008) as


(4) $$F_{DCCA}^{2}\left(s\right)=\frac{1}{N-s+1}\sum_{j=1}^{N-s+1}f_{DCCA}^{2}\left(s,j\right)$$

and its square root as


(5) $$F_{DCCA}\left(s\right)=\sqrt{\frac{1}{N-s+1}\sum_{j=1}^{N-s+1}f_{DCCA}^{2}\left(s,j\right)}.$$

From Eqs. (3) and (5) one can see, that if we consider the special case of *x*(*t*) *y*(*t*), we arrive at the formula of univariate DFA


(6) $$F_{DFA}\left(s\right)=\sqrt{\frac{1}{N-s+1}\sum_{j=1}^{N-s+1}f_{DFA}^{2}\left(s,j\right)},$$

where $f_{DFA}^{2}\left(s,j\right)={\left(s-1\right)}^{-1}\sum_{i=j}^{j+s-1}{\left(X\left(i\right)-{\tilde{X}}_{j}\left(i\right)\right)}^{2}$ (Peng et al., 1994; Podobnik et al., 2011). In case of long-range autocorrelations *F*_*DFA*_(*s*)∝*s*^α^, while in case of long-range cross-correlations *F*_*DCCA*_(*s*)∝*s*^λ^ (Podobnik and Stanley, 2008). From these measures, analogously to the Pearson cross-correlation coefficient Zebende (2011) defined the DCCA cross-correlation coefficient for non-stationary time series as


(7) $$\rho_{DCCA}\left(s\right)=\frac{F_{DCCA_{x,y}}^{2}\left(s\right)}{F_{DFA_{x}}\left(s\right)F_{DFA_{y}}\left(s\right)},$$

where *F*_*DFA*_*x*__(*s*) and *F*_*DFA*_*y*__(*s*) denote the univariate scaling functions of *x*(*t*) and *y*(*t*), with *F_DCCA_x,y__*(*s*) as their bivariate scaling function. It can be shown both empirically and theoretically that ρ_*DCCA*_(*s*) is a dimensionless coefficient ranging from −1 to 1, for which appropriate confidence intervals can be constructed (Podobnik et al., 2011).

Note that the approaches described in Eqs. (1–7) utilize a sliding window approach, while it is more common to divide the analyzed signal into ⌊*N*/*s*⌋ non-overlapping windows at each successive scale *s*, where ⌊⋅⌋ denotes the floor function (Eke et al., 2002; Kantelhardt et al., 2002). Furthermore, the window size *s* is not increased in a linear but rather in a logarithmic fashion, given that the power-law scaling relationship is assessed following log-log transformation of the scaling function and the scale. Hence, logarithmically increasing *s* yields an even sampling of the scale-free measure after the transformation. The most commonly used values of *s* are powers of 2 (i.e., dyadic), usually ranging from 2^3^ to *N*/5 or *N*/4 (Barunik and Kristoufek, 2010; Mukli et al., 2015).

### Online Analysis

When one would like to obtain an estimate on either α, λ, or ρ_*DCCA*_(*s*), one has to run through the signal at each window size at least once. Also, the statistical measure is usually computed using a two-pass formula. However, as shown by Hartmann et al. (2013), one can compute the statistical measure in real-time using a one-pass approach by accumulating data in appropriate helper variables. In what follows we show that the same approach can be utilized in the bivariate case.

#### Analysis Strategy

The key point of the online algorithm is to define helper variables that accumulate the incoming signal datapoint-by-datapoint. Let us consider an incoming stream of two processes, *x*(*t*) and *y*(*t*) and their respective cumulatively summed versions *X*(*t*) and *Y*(*t*). *X*(*t*) and *Y*(*t*) are obtained in real time by simple addition of the next incoming datapoint of *x*(*t*) and *y*(*t*). Then, define the following set of helper variables for each value of *s*:


(8) $$sx\left(s\right)=\sum_{i=t}^{t+s-1}X\left(i\right),sy\left(s\right)=\sum_{i=t}^{t+s-1}Y\left(i\right)$$

$$sxi\left(s\right)=\sum_{i=t}^{t+s-1}iX\left(i\right),syi\left(s\right)=\sum_{i=t}^{t+s-1}iY\left(i\right)$$

$$sx^{2}\left(s\right)=\sum_{i=t}^{t+s-1}X^{2}\left(i\right),sy^{2}\left(s\right)=\sum_{i=t}^{t+s-1}Y^{2}\left(i\right)$$

$$sxy\left(s\right)=\sum_{i=t}^{t+s-1}X\left(i\right)Y\left(i\right).$$

**FIGURE 1:**
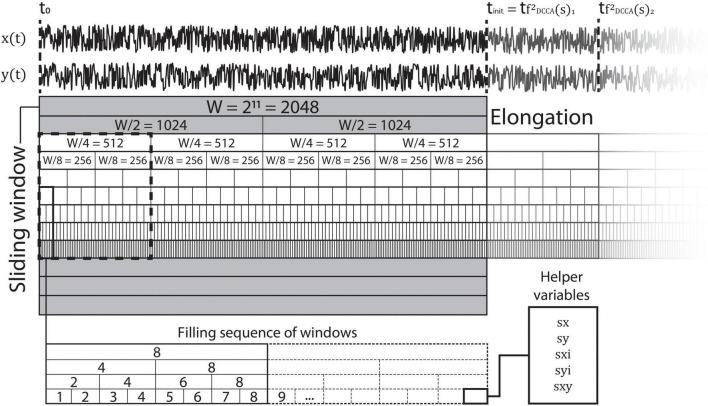
Methodology of real-time parallel signal processing. The sliding window (containing multiples of each given window size) is continuously filled with values from each time series in a manner shown in the box at the bottom. An estimate of the scaling function is first computed at *t*_*init*_ when the sliding window is completely filled. Subsequently, the window slides forward by a length of *s*_*max*_. The shaded window scales are left unused as advised by previous studies (Peng et al., 1994).

Notably, these helper variables are defined for each scale (window size) *s*, and are used to fill the values of $f_{DCCA}^{2}\left(s\right)$ in a fashion which is both parallel and sequential, as illustrated in Figure 1. Precisely, helper variables are initiated at all scales, then once a given window at scale *s* is “filled,” the scaling function value is computed and stored, and the corresponding helper variables at scale *s* are re-initialized. Note that helper variables *sx*^2^(*s*) and *sy*^2^(*s*) are only defined here explicitly for illustrative purposes [see Eq. (12) below] but are not required for real-time DCCA analysis.

As an example, at the beginning of the analysis helper variables are zero at all scales and start to accumulate data points as they arrive. Given a dyadic set of scales ranging from *s*_*min*_ = 2^3^, the first window will be filled after eight datapoints. At that timepoint, $f_{DCCA}^{2}\left(8,1\right)$ is computed (see below) and stored, and *sx*(8), *sy*(8), *sxi*(8), *syi*(8), and *sxy*(8) are re-initialized to 0 and start accumulating data again at *t* = 9. The next time when another window is filled is at *t* = 16, however in this case both windows at *s* = 2^3^ and *s* = 2^4^ are filled. Therefore, both $f_{DCCA}^{2}\left(8,2\right)$ and $f_{DCCA}^{2}\left(16,1\right)$ are computed and stored, and their corresponding helper variables re-initialized. The procedure continues in a similar fashion until windows of all sizes are filled, and then it starts over again. From this example it follows, that one also has to define two index variables as well: a relative index *i* for each scale *s*, which denotes the offset in the current windows of all sizes [which is required for obtaining *sxi*(*s*) and *syi*(*s*)] and a window index *w* that points to the actual largest scale (i.e., if the analysis is at relative data point 512 in *W*, then the algorithm computes $f_{DCCA}^{2}\left(s,j\right)$ values up at scales 2^3^ − 2^9^, thus *w* = 7). The offset index *i* is reset at each scale when the current window is filled at the given scale, while the window index *w* is reset each time when the largest window (*s*_*max*_) is filled. Note that in the real-time algorithm *W* denotes the moving analysis window size, i.e., the resolution of the analysis. Also note that this moving window is progressed by steps of *s*_*max*_ and the elongation is circular, meaning that once the window progressed, scaling function values from the first *s*_*max*_ data points are dropped, and the rest is shifted backwards (see Figure 1). Then, it also follows that initially the algorithm also has to store the number of times the window at *s*_*max*_ is filled, as to return the first estimate of $F_{DCCA}^{2}\left(s\right)$ when *W* datapoints are processed, and then provide the new estimate after every *s*_*max*_ datapoints. Finally, the algorithm is memory efficient since it does not have to store the entire signal of length *W* to compute the scaling function, only the helper variables at every window sizes.

#### Online Detrending

In order to $f_{DCCA}^{2}\left(s,j\right)$ one first has to compute the local trend in window (*s*, *j*) and then subtract it from the given segment of data [see Eq. (3)]. This procedure is usually carried out by least squares estimation of the trend after the data segment is accumulated. Nevertheless, with substituting the appropriate helper variables from Eq. (8) to Eq. (2) one can obtain both coefficients of a linear trend for a segment of *X*(*t*) in real time as


(9) $$m_{x,s}=-\frac{6\left(-2\sum_{i=1}^{s}iX\left(i\right)+\left(n+1\right)\sum_{i=1}^{n}X\left(i\right)\right)}{s\left(s^{2}-1\right)}=-\frac{6\left(-2sxi\left(s\right)+\left(n+1\right)sx\left(s\right)\right)}{s\left(s^{2}-1\right)}$$

$$b_{x,s}=\frac{\sum_{i=1}^{s}X\left(i\right)}{s}-\frac{m_{x,s}\left(s+1\right)}{2}=\frac{sx\left(s\right)}{s}-\frac{m_{x,s}\left(s+1\right)}{2},$$

and similarly, for *Y*(*t*).

#### Online Derivation of the Measures

We can also reformulate the general formula for $f_{DCCA}^{2}\left(s\right)$ from Eq. (3) as


(10) $$f_{DCCA}^{2}\left(s\right)=\frac{1}{s}\sum_{i=1}^{s}\left(X\left(i\right)-\tilde{X}\left(i\right)\right)\left(Y\left(i\right)-\tilde{Y}\left(i\right)\right)=\frac{1}{s}\sum_{i=1}^{s}\left(X\left(i\right)-m_{x,s}\times i-b_{x,s}\right)\left(Y\left(i\right)-m_{y,s}\times i-b_{y,s}\right).$$

Note, that all the terms in Eq. (10) can indeed be expressed via helper variables defined in Eq. (8), so we arrive to the real-time formula for $f_{DCCA}^{2}\left(s\right)$ as


(11) $$f_{DCCA}^{2}\left(s\right)=\frac{1}{s}sxy\left(s\right)-\frac{m_{y,s}}{s}sxi\left(s\right)-\frac{b_{y,s}}{s}sx\left(s\right)-\frac{m_{x,s}}{s}syi\left(s\right)-\frac{b_{x,s}}{s}sy\left(s\right)+\frac{m_{x,s}m_{y,s}s^{2}}{3}+\frac{m_{x,s}m_{y,s}s}{2}+\frac{m_{x,s}m_{y,s}}{6}+\frac{m_{x,s}b_{y,s}\left(s+1\right)}{2}+\frac{m_{y,s}b_{x,s}\left(s+1\right)}{2}+b_{x,s}b_{y,s},$$

which is one of the main contributions of this manuscript. It can also be shown that if we take the special case of *X* (*t*) = *Y* (*t*), Eq. (11) reduces to


(12) $$f_{DFA}^{2}\left(s\right)=\frac{1}{s}sy^{2}\left(s\right)-\frac{2m_{y,s}}{s}syi\left(s\right)-\frac{2b_{y,s}}{s}sy\left(s\right)+\frac{m_{y,s}^{2}s^{2}}{3}+\frac{m_{y,s}^{2}s}{2}+\frac{m_{y,s}^{2}}{6}+m_{y,s}b_{y,s}\left(s+1\right)+b_{y,s}^{2},$$

which is equivalent to the real-time DFA formula presented as Eq. (15) of Hartmann et al. (2013) after taking the square root. After $f_{DCCA}^{2}\left(s,j\right)$ is obtained for all windows *j* at all scales *s* one can obtain $F_{DCCA}^{2}\left(s\right)$ by averaging $f_{DCCA}^{2}\left(s,j\right)$ at all scales over *j*. Note that the algorithm does not have to explicitly store all $f_{DCCA}^{2}\left(s,j\right)$ for different values of *j*; once a window is filled $f_{DCCA}^{2}\left(s,j\right)$ is just added to $f_{DCCA}^{2}\left(s\right)$, and once all windows are filled $f_{DCCA}^{2}\left(s\right)$ is divided by ⌊*N*/*s*⌋ for the corresponding scale. This denominator term depends only on arbitrarily defined parameters (the analysis window and set of scales) and thus can be defined upon initialization.

#### Matrix Notation Formula

The above formulas were derived assuming two simultaneous processes, however in real-world situations one often has to deal with a larger set of signals, e.g., electroencephalography (EEG) recorded from many cortical regions or a portfolio of various stocks. In such a case, computing $f_{DCCA}^{2}\left(s\right)$ sequentially in a pairwise manner between the possible pairs of processes rapidly becomes increasingly inefficient as the number of processes grows. Therefore, it is desired to re-formulate Eq. (11) in matrix notation that allows for computing not only all pairwise interactions at the same time, but the univariate scaling functions of each process as well.

We start with the helper variables of Eq. (8) and define *n*_*ch*_ and *n_s_* as the number of processes (channels) and the number of scales *s*, respectively. In the pairwise case helper variables could be represented as 1 × *n*_*s*_ arrays for *x*(*t*) and *y*(*t*). Let us consider a multivariate process $\bar{x}\left(t\right)$ with *K* elements


$$\bar{x}\left(t\right)=\left[\begin{array}{c} x_{1}\left(t\right)\\ x_{2}\left(t\right)\\ \vdots \\ x_{K}\left(t\right) \end{array}\right]$$

and its cumulatively summed version $\bar{X}\left(t\right)$


$$\bar{X}\left(t\right)=\left[\begin{array}{c} X_{1}\left(t\right)\\ X_{2}\left(t\right)\\ \vdots \\ X_{K}\left(t\right) \end{array}\right]$$

In this case we can collapse the corresponding helper variables onto *n*_*ch*_ × *n*_*s*_ arrays as


$$\bar{sx}\left(k,s\right)=\sum_{i=t}^{t+s-1}X_{k}\left(i\right),k=1,\ldots ,K$$

(13) $$\bar{sxi}\left(k,s\right)=\sum_{i=t}^{t+s-1}iX_{k}\left(i\right),k=1,\ldots ,K$$

and the second order term to a *n*_*ch*_ × *n*_*ch*_ matrix as


(14) $$\bar{sx^{2}}\left(s\right)=\sum_{i=t}^{t+s-1}\bar{X}\left(i\right)\bar{X}{\left(i\right)}^{T},$$

where $\bar{X}{\left(i\right)}^{T}$ is the transpose of $\bar{X}\left(i\right)$. *sx*^2^(*s*) is then broadcasted to a *n*_*ch*_ × *n*_*ch*_ × *n*_*s*_ array. Linear trend-fitting in each window can be performed simultaneously for all signals by representing them as vectors:


$${\bar{m}}_{s}=-\frac{6\left(-2\sum_{i=1}^{s}i\bar{X}\left(i\right)+\left(n+1\right)\sum_{i=1}^{n}\bar{X}\left(i\right)\right)}{s\left(s^{2}-1\right)}=-\frac{6\left(-2sxi\left(\vdots ,s\right)+\left(n+1\right)sx\left(\vdots ,s\right)\right)}{s\left(s^{2}-1\right)}$$

(15) $${\bar{b}}_{s}=\frac{\sum_{i=1}^{s}\bar{X}\left(i\right)}{s}-\frac{{\bar{m}}_{s}\left(s+1\right)}{2}=\frac{sx\left(\vdots ,s\right)}{s}-\frac{{\bar{m}}_{s}\left(s+1\right)}{2},$$

where the symbol ⋮ denotes values from all rows (channels) at column (scale) *s*. Finally, one has to rewrite Eq. (11) by using Eqs. (13–15) to obtain the real-time DCCA formula in matrix notation as


(16) fDCCA2¯(s)=1n[sx2¯(s)-[m¯ssxi¯(⋮,s)T+[m¯ssxi¯(⋮,s)T]]T-[b¯ssx¯(⋮,s)T+[(b¯ssx¯(⋮,s)T)]T]]+(s+1)(2s+1)6m¯sm¯sT+s+12[m¯sb¯sT+[m¯sb¯sT]T]+b¯sb¯sT,

which is the other important contribution of the presented work. Given that $\bar{f_{DCCA}^{2}}\left(s\right)$ has to be obtained for every scale *s*, the values are eventually stored in an arrays of size *n*_*ch*_×*n*_*ch*_×*n*_*s*_ for each sub-window of size *s*_*max*_ (i.e., once a window of size *s*_*max*_ is filled). Storing these in separate arrays is required for the circular elongation of the analysis window of size *N*. Finally, one obtains $F_{DCCA}^{2}\left(i,j,s\right)$, an array of size *n*_*ch*_ × *n*_*ch*_ × *n*_*s*_ (with *i*, *j* = 1, …, *n*_*ch*_ and *s* = 1, … , *n*_*s*_) after collecting $\bar{f_{DCCA}^{2}}\left(s\right)$ from *N* datapoints, sum them up and divide them with an appropriate constant, which similarly to the bivariate case only depends on *s* and *N* and thus can be defined at initialization.

The obtained array $F_{DCCA}^{2}\left(i,j,s\right)$ has several important properties. First, elements in the main diagonal (i.e., *ij*) are apparently values of the DFA scaling functions of each individual process, namely $F_{DCCA}^{2}\left(i,i,s\right)=F_{DFA,i}^{2}\left(s\right)$ of process *i*. On the other hand, non-diagonal cells store the *F*_*DCCA*_ values of the corresponding processes. Second, one can simply obtain the DFA scaling exponent α in real time, as from Eq. (16) it can be seen that elements in the main diagonal are always positive, therefore one can take the square root, perform the logarithmic transformation and obtain α *via* least squares regression on log(*s*). On the contrary, one cannot take the square root of non-diagonal elements, as their values are not guaranteed to be non-negative, and thus a real-time assessment of the DCCA scaling exponent λ is not possible with this algorithm. However, given that both $F_{DCCA}^{2}\left(i,j,s\right)$ and $F_{DFA,i}^{2}\left(s\right)$ are obtained for all *i*, *j* and *s* in real-time, one can simply transform the $F_{DCCA}^{2}\left(i,j,s\right)$ array into a ρ_*DCCA*_(*i*, *j*, *s*) array according to Eq. (7).

### *In silico* Experiments

#### Simulated Datasets

In order to evaluate the performance of our real-time algorithm we generated time series pairs with known long-range cross-correlations using the mixed-correlated autoregressive fractionally integrated moving average (mc-ARFIMA) framework of Kristoufek (2013). A single ARFIMA process with parameter *d* is defined as


(17) $$x_{t}=\sum_{n=1}^{\infty }a_{n}\left(d\right)x_{t-n}+\varepsilon_{t},$$

where *n* is the time scale, *d* ∈ (0; 0.5), ε_*t*_ are independent and identically distributed Gaussian random variables with zero mean and unit variance, and the weights *a*_*n*_(*d*) are defined as $a_{n}\left(d\right)=\frac{d\Gamma \left(n-d\right)}{\Gamma \left(1-d\right)\Gamma \left(n+1\right)}$ where Γ denotes the Gamma function (Podobnik et al., 2008). Importantly, an ARFIMA process expresses long-range autocorrelations with a scaling exponent equal to α = 0.5 + *d*. The mc-ARFIMA framework consists of a pair of time series *x_t_* and *y_t_*, each being a linear combination of two independent simple ARFIMA processes so that:


$$x_{t}=w_{1}\sum_{n=1}^{\infty }a_{n}\left(d_{1}\right)x_{1,t-n}+\varepsilon_{1,t}+w_{2}\sum_{n=1}^{\infty }a_{n}\left(d_{2}\right)x_{2,t-n}+\varepsilon_{2,t}$$

(18) $$y_{t}=w_{3}\sum_{n=1}^{\infty }a_{n}\left(d_{3}\right)y_{1,t-n}+\varepsilon_{3,t}+w_{4}\sum_{n=1}^{\infty }a_{n}\left(d_{4}\right)y_{2,t-n}+\varepsilon_{4,t},$$

where

⟨ε_*i*,*t*_⟩ = 0 for *i* = 1, 2, 3, 4

$\left\langle \varepsilon_{i,t}^{2}\right\rangle =\sigma_{\varepsilon_{i}}^{2}$ for *i* = 1, 2, 3, 4

⟨ε_*i*,*t*_ε_*j*,*t*−*n*_⟩ = 0 for *n* ≠ 0 and *i*, *j* = 1, 2, 3, 4

⟨ε_*i*,*t*_ε_*j*,*t*_⟩ = ρ_*ij*_ for *i*, *j* = 1, 2, 3, 4 and *i* ≠ *j*, (i.e., ρ_*ij*_ captures the covariance of the noise terms).

It can be shown that with appropriate parameter settings *x_t_* and *y_t_* expresses true long-range cross correlations with $\lambda =\frac{d_{2}+d_{3}+1}{2}$ (Kristoufek, 2013). Unless it is stated otherwise, time series were simulated with parameter settings *w*_1_ = *w*_4_ = 0.2, *w*_2_ = *w*_3_ = 1, *d*_1_ = *d*_4_ = 0.4, *d*_2_ = *d*_3_ = 0.3, and $\sigma_{\varepsilon_{i}}^{2}=1$ for *i* = 1, 2, 3, 4 and ρ_2,3_ = 0.9 (with ρ_*ij*_ = 0 for all other values of *i*, *j*) to emphasize long-term cross persistence, following the recommendations of Kristoufek (2013).

#### Execution Time

Time series pairs were simulated with varying length *N* ranging from 2^8^ to 2^17^. To ensure statistical stability, 1,000 realizations of mc-ARFIMA processes were generated at every length, and the execution times were stored. For the sake of simplicity, we set the analysis window *W* equal to the signal length *N* in the real-time analysis. The scales (window sizes) were varied according to the signal length. Further details of the runtime analysis are shown in Table 1.

**TABLE 1:** Execution times for the offline and online implementations of detrended cross-correlation analysis (DCCA).

*N*	Offline (s)	Online (s)	s_min_	s_max_	w
2^8^	0.1252	0.0004	2^2^	2^6^	5
2^9^	0.2277	0.0005	2^2^	2^7^	6
2^10^	0.4308	0.0009	2^2^	2^8^	7
2^11^	0.3974	0.0015	2^3^	2^9^	7
2^12^	0.8930	0.0029	2^3^	2^10^	8
2^13^	1.4896	0.0053	2^3^	2^10^	8
2^14^	1.5242	0.0099	2^4^	2^11^	8
2^15^	3.0784	0.0199	2^4^	2^11^	8
2^16^	6.2421	0.0369	2^4^	2^12^	9
2^17^	13.3261	0.0729	2^4^	2^12^	9

*Runtimes were averaged over 1,000 runs and presented in seconds. The analysis window size W was set equal to signal length N, s_min_ and s_max_ denote the minimal and maximal scales, and w denotes the number of window sizes.*

#### Precision

Precision of the online algorithm was tested via the detrended cross-correlation coefficient DCCC at three different signal lengths *N* = 2^8^, 2^10^, and 2^12^. Parameters *d_1_* and *d_4_* were collectively set to 0.45, while *d_2_* and *d_3_* (controlling λ) were varied from 0.05 to 0.45 in 0.05 increments. For every case 100 realizations of mc-ARFIMA processes were generated. In the online analysis *W* was again set equal to the signal length *N* and the analysis scales were set as shown in Table 1. The error of the online algorithm compared to the baseline offline algorithm was captured as the mean squared error (MSE) computed over the analysis scales, according to


(19) $$MSE\left(N\right)=\frac{1}{n_{s}}\sum_{s=s_{min}}^{s_{max}}{\left(\rho_{DCCA,offline}\left(s\right)-\rho_{DCCA,online}\left(s\right)\right)}^{2},$$

where *n_s_* denotes the number of scales at signal length *N*, and ρ_*DCCA*,*offline*_(*s*) and ρ_*DCCA*,*online*_(*s*) denotes the DCCC values at scales *s* obtained with the offline and online algorithms, respectively. In the case of ρ_*DCCA*,*offline*_(*s*), *F*_*DFA*_*x*__(*s*), and *F*_*DFA*_*y*__(*s*) [see Eq. (7)] were obtained with offline implementation of DFA.

#### Pairwise Versus Matrix Implementation

In case of two time series although the matrix notation formula provides a straightforward way to obtain the bivariate scaling function, it has higher memory requirements. As the number of parallel time series *n* becomes larger, the memory requirement grows with *n*^2^, increasing execution time, however the number of operations remains the same. In comparison, if one computes the bivariate scaling function in a pairwise fashion the memory usage remains unaltered, while the number of operations grows with *n*^2^. Consequently, the execution time is expected to increase with the number of processes to analyze in a slower tendency in case of the matrix when compared to the pairwise implementation. Therefore, we investigated the effect of the number of processes *n* on the execution time at three different signal lengths *N* = 2^8^, 2^10^, and 2^12^. Given that the obtained matrix of scaling functions $\bar{f_{DCCA}^{2}}\left(s\right)$ is symmetric along the main diagonal (i.e., $\bar{f_{DCCA}^{2}}{\left(s\right)}_{i,j}=\bar{f_{DCCA}^{2}}{\left(s\right)}_{j,i}$), in the pairwise implementation ρ_*DCCA*_(*s*) of processes *i* and *j* was computed only once. The number of processes were increased from *n* = 2 to *n* = 20, with 100 realizations of mc-ARFIMA processes in each case.

#### Effect of Additive Noise and Spike Artifacts

Even though the robustness of offline DFA and DCCA implementations against noise components have been assessed by previous studies (Ludescher et al., 2011; Delignieres and Marmelat, 2014; Nagy et al., 2017), it is imperative that we also briefly assess the performance of the proposed online algorithm under such conditions. Here we consider two general scenarios: (i) the presence of additive white noise and (ii) the presence of spike-like artifacts common in physiological and other empirical signals. In all the following cases, mc-ARFIMA time series pairs of length *N* = 2^12^ were generated with *d_2_* and *d_3_* varying between 0.05 and 0.45 in 0.05 increments. For all parameter settings *n* = 100 time series pairs were simulated, and the performance of the algorithm was assessed via the MSE similarly to Eq. (19), only in these cases the differences were computed between ρ_*DCCA*_ obtained from the raw (noise-free) and contaminated versions of the same time series pairs. The level of additive white noise contamination was set by the signal-to-noise ratio (SNR), with SNR defined as the ratio of the variance of the original signal and the variance of the added noise component. We considered three different levels of contamination with SNR of 100, 10, and 1. In the second scenario we considered three different cases of contamination by spike-like components: only one of the signals contains artifacts (Type A), both signals contain independent artifacts (Type B), and both signals contain the same artifact components (Type C). In each case, 10 randomly positioned, non-overlapping, Hanning-type spike components of width 20 datapoints were added to the given time series. The amplitude of the spikes was set to be four times the variance of the original time series.

### Application to Empirical Data

To illustrate the real-world applicability of our algorithm, we analyzed EEG recordings obtained in eyes open (EO) and eyes closed (EC) conditions. The matrix implementation of our online algorithm was utilized to obtain the DCCC values between cortical region pairs and the DFA scaling exponents of individual cortical regions. A classifier was then trained to automatically distinguish between EO and EC conditions based on DCCC and α values, as to illustrate the potential future utility of real-time DCCA analysis in automated mental state monitoring.

#### Dataset

As a demonstration, we analyzed electroencephalographic recordings of young, healthy volunteers. A total of 25 participants were recruited who underwent 6 min of continuous EEG monitoring in resting condition with the first and second 3-min epochs in EC and EO states, respectively. Measurements were carried out in a dark room and participants were seated in a comfortable armchair in front of a computer screen. During the EO period, participants were instructed to focus their vision on a presented fixation cross to reduce eye movements. During both conditions subjects were asked to relax, disengage in their minds, and refrain from movement as much as possible. Three participants were later excluded for bad signal quality, resulting in a final number of 22 participants (12 female, aged 23.9 ± 2.3 years, 20 right-handed). All participants provided written informed concept prior to the measurement and the study was approved by the Regional and Institutional Committee of Science and Research Ethics of Semmelweis University (approval number: 2020/6) in accordance with the Declaration of Helsinki.

Recordings were carried out using an Emotive Epoc + v1.1 wireless EEG device (Emotiv Systems Inc., San Francisco, CA, United States) with the corresponding EmotivPRO software. Neural activity was recorder from 14 cortical regions according to the international 10–20 standard montage (AF3, AF4, F3, F4, F7, F8, FC5, FC6, T7, T8, P7, P8, O1, and O2). Impedances were kept under 20 kΩ and the data was digitalized at a 256 Hz temporal resolution.

#### Data Analysis

Data was internally filtered by the Emotiv EPOC + device between 0.2 and 45 Hz with a built-in 5th order digital Sync filter, with additional digital notch filtering at 50 and 60 Hz. Although the analysis was carried out offline, in order to mimic an online analysis framework, we did not apply any additional artifact removal techniques apart from visually inspecting the obtained datasets (which resulted in the exclusion of the data from three subjects, as mentioned previously). The analysis window *W* was set to *W* = 512 datapoints (2 s) with scales ranging from *s*_*min*_ = 2^3^ to *s*_*max*_ = 2^7^ (*w* = 5). Thus, a new estimate of DCCC was obtained at every 2 s. Additionally, given that *F*_*DFA*_(*s*) was also obtained, we computed the DFA scaling exponents for all channels as well and included them as features for classification (see section “Classification”).

#### Classification

The classification pipeline was implemented in Python 3.7.4 (Jupyter Notebook developing environment), using functions of the scikit-learn package (version 0.24.2). We used a Linear classifier with Stochastic Gradient Descent training (SGDClassifier) with elastic net (Zou and Hastie, 2005) penalty to facilitate sparsity of features. In the training procedure we performed a subject based random train-test split of the data (i.e., all samples from the same subject was assigned either to the train or test sets, exclusively) to an approximate 25–75 ratio (5 test and 17 train subjects). A grid search with five-fold cross-validation was carried out using data only from the training set to identify the best hyperparameters of the model, *alpha* and the *l1_ratio*. Then, the final model was initialized with the best hyperparameter settings (*alpha* = 0.01, *l1_ratio* = 0.3), trained on the entire training set and evaluated on the previously untouched test set. The features used for classification consisted of DCCC coefficients of all channel pairs obtained at all five scales, as well as channel-wise DFA scaling exponents. Model performance was identified standard metrics such as accuracy, precision, and recall (sensitivity) and F1-score (Fawcett, 2006).

### Computational Environment

The online algorithms were developed in Matlab (The Mathworks, Natick, MA, United States). *In silico* simulations, tests and analysis of EEG data were carried out on a personal computer with an Intel Core i7-6600U CPU 2.60 GHz processor using Matlab. The classification framework was developed and implemented in Python. Implementations of the algorithm will be made available soon at the repository at https://github.com/samuelracz/rsDCCA.

## Results

### Execution Time

Table 1 summarizes the results regarding runtime of the online and offline algorithms for DCCA. Execution times were for the real-time algorithm were approximately three orders of magnitude smaller than those of the offline method. For both pipelines we found execution time to be a linear function of the window size *N*, although with substantially different constants (9.79 × 10^−5^ vs. 5.54 × 10^−7^).

### Precision

**FIGURE 2:**
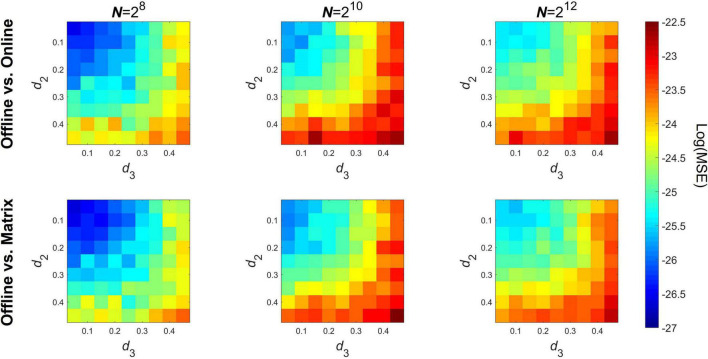
Precision of the online algorithms compared to the offline implementation. The top row shows the logarithm of mean squared error when compared between the offline and the pairwise online implementation, while the bottom row shows the same in case of the matrix notation formula. Columns show the results obtained at different signal lengths. MSE, mean squared error; *N*, signal length.

The precision of both the pairwise and matrix formulas were tested against baseline values obtained with the offline implementation. The 10-based logarithm of the MSE of DCCC is shown on Figure 2 for three different signal lengths, where the upper and lower panels show MSE of the pairwise the matrix implementations, respectively. As seen, errors range below 10^–22^, thus it can be concluded that the online algorithms return practically the same estimates as the offline implementation. Notably, two tendencies are apparent; first, MSE slightly increases with signal length and second, MSE increases as the parameter *d* of either (and/or both) time series increases. MSE of the pairwise and matrix implementations were nearly equal to each other.

### Pairwise Versus Matrix Implementation

**FIGURE 3:**
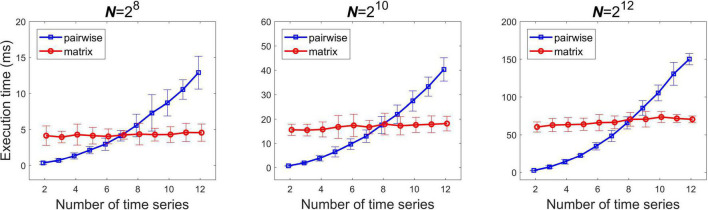
Comparison of the execution times of the pairwise and matrix implementations of the real-time algorithm. The three panels show results obtained from different signal lengths. *N*, signal length.

Runtime was evaluated in case of multiple time series when computing all pairwise estimates with the matrix formula and with applying the pairwise formula sequentially. Figure 3 shows the analysis carried out at three different signal lengths. As expected, execution time grew according to *n*^2^ for the sequential pairwise pipeline, while it increased roughly linearly with the matrix pipeline (in the investigated range). At low channel numbers the pairwise algorithm proved more cost-effective. Execution times were roughly the same for the two implementations in case of 7, 8 and eight time series for *N* = 2^8^, 2^10^, and 2^12^ respectively, while over these the matrix algorithm performed better.

### Effect of Noise

**FIGURE 4:**
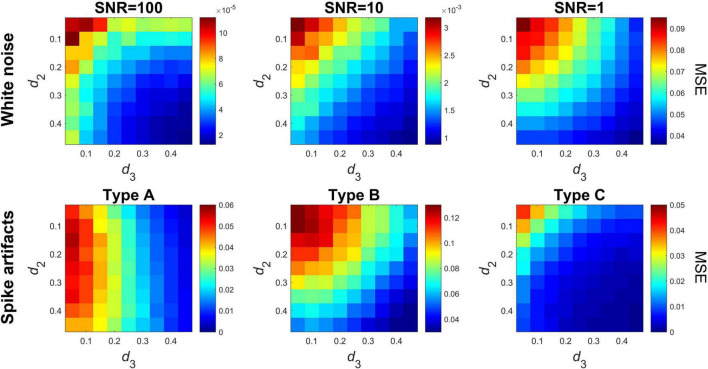
Effect of additive white noise and spike artifacts. The upper row shows the mean squared error between clean and white noise-contaminated time series at three different levels of signal-to-noise ratio. The bottom row shows the mean squared error for time series before and after addition of spike-like artifacts. Three cases are shown; in Type A (left) only one of the time series contains artifacts, in Type B (middle) both time series contain artifacts although independent of each other, while in Type C (right) both time series contain the same artifacts. MSE, mean squared error; SNR, signal-to-noise ratio.

The algorithm proved robust against the presence of additive white noise (Figure 4, upper panel), with MSE values in the range of 10^−5^ and 10^−3^ except for the case of SNR = 1. As expected, performance decreased with decreasing SNR. Spike artifacts had a slightly more pronounced effect, especially with *d_2_* and *d_3_* close to zero (Figure 4, lower panel). Type A spikes had a moderate effect on ρ_*DCCA*_ (MSE below 0.06) when *d*_3_ < 0.2, however MSE reduced to practically zero with increasing *d_3_*. Note that the asymmetry is due to the fact that spikes were always added to the first time series (with parameter *d_2_*). Spike artifacts had the most prominent effect in case Type B (i.e., independent spike artifacts in both signals), especially at small *d_2_* and *d_3_* values. The effect diminished with increasing *d_2_* and *d_3_*. Finally, Type C spikes (synchronized spikes in both signals) had the less effect, with MSE over 0.025 only when both *d_2_* and *d_3_* were below 0.1.

### Experimental Electroencephalography Data

Details of classifier performance are shown in Table 2. The classifier exceeded 80% accuracy on the training set, while it almost reached 78% on the test set. This implies that the fitted model was biased, however it could also be characterized with a low variance, with the test accuracy not being substantially worse than that on the training set.

**TABLE 2:** Performance of the classifier.

	Training set	Test set
Accuracy	80.64%	77.99%
Precision	78.20%	74.28%
Recall	84.88%	85.53%
F1-score	81.41%	79.51%

**FIGURE 5:**
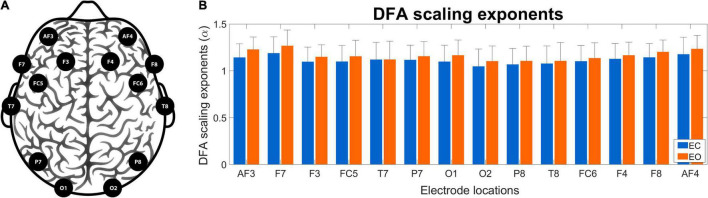
Electrode localizations and DFA scaling exponents. Panel **(A)** shows the channel layout of the Emotiv Epoc + wireless EEG device and the corresponding cortical regions. Panel **(B)** shows the obtained regional DFA scaling exponents in eyes-closed (blue) and eyes-open (orange) conditions. The obtained exponents appear similar in the two states, with a weak tendency of higher values in the EO condition. DFA, detrended fluctuation analysis; EEG, electroencephalography; EC, eyes-closed; EO, eyes-open.

**FIGURE 6:**
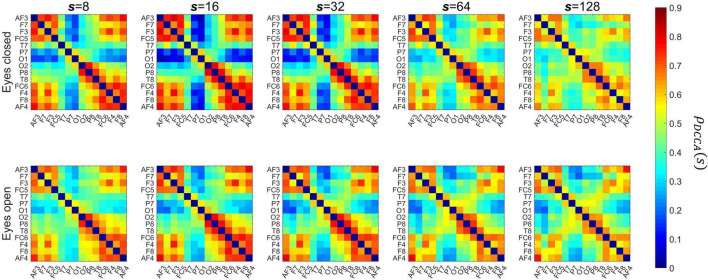
Grand-average matrices of detrended cross-correlation coefficients. The columns show the matrices for various scales, with the top and bottom rows presenting results in eyes closed and eyes open conditions, respectively. *s*, scale.

Figure 5 shows the channel layout for the EEG system (panel A) and the grand average of DFA scaling exponents in the two conditions (panel B). The α values appear higher in EO than in EC condition, as well as a weak topological organization can be observed with higher scaling exponents over frontal regions when compared to occipital regions in both states. Figure 6 shows the grand average of DCCC matrices obtained in the two states, at the various scales. It can be seen that on the shorter scales there are two clear clusters corresponding to the left and right frontal cortex that are also strongly connected to each other, while fronto-occipital connections are generally weaker, with DCCC often close to zero. This topology becomes less prominent on larger scales, where both connectivity of the frontal regions decreases while at the same time fronto-occipital connections become slightly stronger. A smaller occipital module can also be seen, which shows lateralization to the right hemisphere. The same topological organization can be found both in EC and EO states, although at shorter scales fronto-occipital connections appear stronger in EO when compared to EC.

## Discussion

In this work we introduced two real-time implementations for obtaining the bivariate DCCA scaling function of long-term coupled time series. The first formula is a direct generalization of the online formula for obtaining the DFA scaling function presented by Hartmann et al. (2013) to the bivariate domain. More importantly, we showed that this formula can also be expressed in matrix operations, making it feasible to carry out the bivariate scaling function estimation for more than two processes efficiently. Incidentally this algorithm also returns the univariate DFA scaling functions of the individual processes, thus it can be easily extended to return not only the scaling functions for DCCA but also detrended cross-correlation coefficients.

Most importantly, as rightfully stressed in Hartmann et al. (2013) the presented method is unique as it is by design analyses an incoming stream of data datapoint-by-datapoint, thus allowing for true real-time utility, in contrast to real-time-like applications of offline methods that only built on the relatively short dynamics of data compared to available computational power (i.e., the desired estimates can be computed with an offline algorithm well before the next unit of data is received). Real-time analysis of data is required in many physiological applications, such as monitoring mental workload (Myrden and Chau, 2017; Shafiei et al., 2020), depth of anesthesia (Ha et al., 2018; Park et al., 2020), automated tracking of sleep stages (Michielli et al., 2019), or brain computer interface applications (Banville and Falk, 2016). Specifically, bivariate (or multivariate) analysis of neural recordings is the central concept of functional connectivity (FC) studies (Bastos and Schoffelen, 2016). Although initially FC analyses were carried out primarily in a static manner (i.e., utilizing the recorded neurophysiological signals in their entirety to establish the existence and strength of a functional connection between the investigated brain regions) the notion of dynamic functional connectivity (DFC) – namely to consider the time-varying nature of functional connections in the brain – gained considerable attention lately (Preti et al., 2017). Accordingly, there is a palpable need for methods that can be efficiently used in real-time estimation of FC patterns (Garcia-Prieto et al., 2017). Our algorithm might prove essential in this emerging field not only for its online nature, but also its ability to handle non-stationarity in data – a common feature of EEG recordings –, given that DCCA was originally introduced to capture long-range cross-correlations in non-stationary signals (Podobnik and Stanley, 2008). Furthermore, the DCCA algorithm proved moderately robust against the presence of noise or artifacts common in neurophysiological signals, see Figure 4. Nevertheless, applicability of our presented algorithm is not limited to neuroscience or physiological data in general; indeed, the most common application of DCCA and DCCC analysis is to assess long-range coupling in financial time series (Cao et al., 2014; Zebende and Da Silva, 2018) representing concerted actions in the market. Financial data analysis is therefore yet another discipline of science where real-time applications are common and desirable (Wang et al., 2010; Monteforte and Moretti, 2013), and where our online DCCC algorithm might prove valuable.

In this work we focused specifically on the assessment of the scaling function $f_{DCCA}^{2}\left(s\right)$ itself, as being the most computationally expensive part of the analysis of long-term correlations. Nevertheless, in most cases the goal of such analyses is to obtain the bivariate scaling exponent λ. It has been proposed by Podobnik and Stanley (2008) that *f*_*DCCA*_(*s*) scales as *s*^λ^, or equivalently, $f_{DCCA}^{2}\left(s,j\right)$ scales as *s*^2λ^ (Kristoufek, 2017). The scaling exponent is routinely estimated by ordinary linear regression following log transformation of *f*_*DCCA*_(*s*) and *s*, given that log(*f*_*DCCA*_(*s*))∝ λ ⋅ log(*s*) and thus the scaling exponent is simply the slope of the fitted linear function. Although estimating λ this way is intuitive and simple, it can prove difficult in practice: given that $f_{DCCA}^{2}\left(s\right)$ is obtained as the detrended covariance of the corresponding processes [see Eq. (3)], this quantity can be negative, thus preventing taking its square root [as in Eq. (5)] or performing the log transformation. These steps can only be ensured by modifying the DCCA formula and taking the absolute value of detrended covariance as in (Mukli et al., 2018). However, this modification in turn would no longer allow for the data to be processed datapoint-by-datapoint (as the absolute value of the covariance is not equal to the covariance of absolute values), thus preventing the real-time derivation of λ. Nevertheless, the fact that our matrix notation formula provides the DFA scaling functions of the individual processes in the main diagonal has two consequences: (i) real-time tracking of univariate DFA scaling exponents of all processes is possible in a parallel manner, as well as (ii) one can easily convert $f_{DCCA}^{2}\left(s\right)$ of any pair of signals to ρ_*DCCA*_(*s*) in real time by dividing it by the product of the univariate scaling functions of the corresponding signals.

Here we present online formulas for DCCA analysis, however several other techniques are available for capturing long-range coupling, such as the detrended moving-average cross-correlation analysis (DMCA) (Arianos and Carbone, 2009; He and Chen, 2011) and the height cross-correlation analysis (HXA) (Kristoufek, 2011), or frequency domain methods (Kristoufek, 2014b). In a simulation study it has been found that among multiple estimators, the DCCA method has the most bias, which becomes even more substantial with larger signal lengths (Kristoufek, 2017). Note that for shorter signal lengths, which are the focus of most real-time applications in general, DCCA performed comparably if not better than the DMCA and HXA regarding bias (Kristoufek, 2017). Given that DCCA is the direct generalization of the univariate DFA to the bivariate domain (Podobnik and Stanley, 2008), the online (pairwise) formula was similarly developed from the online implementation of DFA by Hartmann et al. (2013). Developments of plausible online implementations for DMCA and HXA might be also of interest for future research given the aforementioned considerations, however this is beyond the scope of the current study.

### *In silico* Evaluation

As expected, the online algorithms substantially overperformed the offline implementation of DCCA with execution times being approximately three orders of magnitude smaller than in the offline case. Importantly, this did not come at the expense of losing precision, MSE of the online implementations were below the range of 10^–20^ when compared with the offline method.

When evaluating precision, we observed that MSE depended slightly on the signal length. This is most likely due to the different window sizes used during the analyses (see Table 1), as with longer time series lengths we obtained ρ_*DCCA*_ (*s*) for larger scales as well (with *s*_*max*_ = 2^8^ and 2^10^). In line with the increase in bias of DCCA at larger time series lengths observed by Kristoufek (2017), we found that the squared error at larger scales were slightly higher when compared to those at smaller scales, and since MSE was computed as the average taken over all scales, this might explain the slight increase in MSE with growing signal length. We also saw an increase in MSE with increasing *d_2_* and/or *d_3_* parameters of the generating mc-ARFIMA models. This phenomenon might be related to the framework used for simulating time series. Accordingly, although the mc-ARFIMA method constitutes a sophisticated and versatile way of generating long-range cross correlated time series in theory (Kristoufek, 2013), its implementation is subject to finite size effects and numerical instabilities. This becomes evident when considering Eqs. (17) and (18), as the sum runs from *n* = 1 to ∞, as well as precision of weights *a*_*n*_(*d*) deteriorate with the increase of *n* due to the Gamma function. It appears that the mc-ARFIMA framework introduces numerical effects that affect the offline and online formulas in a different manner, and the complete resolve of this issue requires further research. Nevertheless, the additional bias observed in this regard was practically negligible and thus it does not devaluate the online implementations of DCCA and DCCC.

Comparing the execution times at varying number of parallel processes produced unsurprising results: in case of a small number of time series pairs a sequential execution of pairwise computations was more efficient than using the matrix formula, due to the larger physical memory requirements of the latter. On the other hand, increasing the number of time series involved affected the runtime only in a linear, while in a quadratic manner for the matrix and pairwise implementations, respectively. Although in our cases the turning points (when the matrix implementation becomes more favorable) were identified approximately at eight parallel time series, these results cannot be generalized to all processes. We only investigated signal lengths in the range 2^8^–2^12^ with pre-specified scale settings. Nevertheless, the analysis window and scales of interest should be set in line with the actual/desired application and characteristics of the given signals at hand. Therefore, we suggest that one should first run both algorithms with the appropriate settings on a representative test (toy) dataset and then decide on the two implementations (matrix or sequential pairwise) in light of the obtained results.

The online algorithm also proved to be robust against the presence of additive white noise in case of reasonable SNRs. Apparently, the effect of noise was stronger when parameters *d_2_* and *d_3_* were set close to zero. Notably, with *d*0 an ARFIMA process produces time series with α = 0.5 + *d* = 0.5, which is equivalent to white noise (Eke et al., 2002). Therefore, this could explain why in such cases even a lesser amount of noise could disturb the weak long-term correlated structure to a greater extent. Nevertheless, the resulting error is still beyond the error boundaries of the DCCA method itself at acceptable levels of SNR (Kristoufek, 2014a). Spike-like artifacts had a more severe effect on ρ_*DCCA*_ estimates, even though the resulting errors remained reasonably small when *d_2_* and *d_3_* were not too close to zero. This pattern might be explained along similar lines as in case of white noise. The performance only dropped more prominently, when both signals were contaminated by independent spike-like artifacts, which is an apparent shortcoming of the method, even though such a scenario renders online data analysis difficult in general. Surprisingly, the algorithm performed the best in the presence of synchronous spikes, which are the most common in case of neurophysiological signals (such as spikes introduced to EEG due to eye blinks or the electric activity of the heart). Nevertheless, these results highlight the importance of appropriate pre-processing when one analyzes empirical data, regardless of its origin. Even though this is an immensely difficult task in case of online data analysis, given the importance of the issue new methods are developed continuously (Venkata Phanikrishna et al., 2021).

### Experimental Data

For our demonstration we deliberately choose a simple experimental paradigm as to evaluate plausible future utility of real-time DCCC analysis in mental state monitoring. In our analysis we only used the previously described DFA/DCCC features and did not apply any explicit feature selection techniques except for opting with the elastic net penalty, which facilitates sparsity of the feature space (Zou and Hastie, 2005). Our classifier reached a reasonable performance, which most likely could be further improved by the inclusion of traditional features such as band-limited spectral power (Banville et al., 2017). Furthermore, it can be expected that better classification performance could be achieved by an EEG system with better spatial and temporal resolution. Since we used online data for demonstrative purposes, in this analysis we did not apply any elaborate pre-processing of EEG except for band-pass filtering and visual inspection. Therefore, it is likely that some artifactual components remained in the analyzed data, such as eye blinks in the EO condition. Based on our simulation results it can be assumed, however, that such artifacts likely had minimal effect on the performance, also considering that most time series had a DFA scaling exponent higher than 0.5 (mostly in the range of 1 and 1.3). Nevertheless, with more sophisticated data cleansing methods one can expect a better classification performance (Venkata Phanikrishna et al., 2021). We also carried out a *post-hoc* cross validation where the training and testing procedure was carried out with 100 different random train-test splits (subject-based) and found that the average accuracy was found as 69.72 ± 5.08%, which was considerably lower than the initial 77.99%. A one-sample *t*-test could not reject the null hypothesis that the population of accuracies obtained from the 100 cross-validation runs came from a distribution with mean 77.99% (*p* = 0.0508). Nevertheless, this drop could be attributed to the fact that hyperparameters were not adjusted at every train-test split via grid search, but the originally identified settings were used for each initialization.

Our results indicate that long-range connectivity is different in EC versus EO conditions, and this difference mainly affects connections linking the occipital and frontal cortices (see Figure 4). This is not necessarily surprising, as with EO condition one would expect processing of visual input, which is in part involves occipito-frontal projections. These results are also partly in line with those indicating that long-term dynamics of functional brain connectivity might change when transitioning from an eyes-closed to eyes-open state (Racz et al., 2018b; Stylianou et al., 2021). The current results serve as another indicator that long-range and fractal aspects of functional connectivity carry physiological relevance regarding the organization of the brain. It has been shown recently that functional brain connectivity shows long-range correlations even when investigated at the global level (Racz et al., 2018a), nodal level (Racz et al., 2019) or in case of individual connections (Stylianou et al., 2020), and that these properties might change due to cognitive stimulation (Stylianou et al., 2021) or pathological conditions such as schizophrenia (Racz et al., 2020). In that regard, our DCCC algorithm could provide further means for dynamic fractal connectivity analysis and thus enhance the currently evolving field of fractal connectivity studies.

Although FC approaches are quite widely used for assessing various cognitive processes and mental states, connectivity-related features are not commonly used in most brain computer interface (BCI) applications (Banville and Falk, 2016), most likely due to their high computational cost. Furthermore, many studies utilizing FC features mostly resort to simple linear covariance or cross-correlation (Rutkowski et al., 2011), which only provide FC estimates on a pre-defined scale and are susceptible to local non-stationarities that are common in EEG data. Real-time acquisition of DCCC resolves both issues by estimating covariance on multiple time scales, as well as dealing with local non-stationarities by local online detrending. Therefore, we believe our online DCCC algorithm might become a valuable asset for future real-time applications based on EEG and other neurophysiological data.

### Limitations

Finally, we have to address the limitations of the current method. The most obvious shortcoming of the presented algorithms is their inability to provide a real-time estimate on the cross-spectral exponent λ, as mentioned previously. Unfortunately, this is an inherent limitation of the method that cannot be resolved under the currently presented framework and requires further development. Another drawback of the proposed algorithm is that although computation is carried out in a datapoint-by-datapoint fashion, estimates on ρ_*DCCA*_(*s*) are only provided after every *s*_*max*_ datapoints, which limits the field of applicability of the current algorithm. Although the frequency of receiving new estimates can be increased by reducing *s*_*max*_, this comes at a price of also reducing the scaling range/analysis window. A straightforward way to resolve this issue would be to modify the algorithm from the non-overlapping window scheme to that shown in Eq. (4), however this would come at the price of an increase in memory usage by a factor of *s*_*max*_. Additionally, in case of scale-free analysis of empirical signals it is often of concern if the obtained measures indeed reflect an underlying characteristic of the investigated system, or just merely reflect numerical/background noise (Mukli et al., 2015). In order to resolve this issue, surrogate data testing is often performed to complement such analyses (Ivanov et al., 1999; Kantelhardt et al., 2002; Racz et al., 2020; Stylianou et al., 2021), however generating surrogates requires the signals in their entirety and thus not a valid solution in case of online analysis. In case of DCCC, one can also adopt the framework proposed by Podobnik et al. (2011) to construct confidence intervals and thus gain insight on the significance of the obtained coefficients. However, given that our main goal here was to provide online formulas for obtaining the DCCCs themselves, we did not include this step and thus it requires future work. Nevertheless, in case of real-world applications *post-hoc* offline surrogate testing of the obtained data might be of desire in order to avoid arriving at false conclusions.

## Conclusion

Here we introduced two real-time formulas for obtaining the DCCA scaling function in real-time. Our formulas vastly overperformed the offline implementation of DCCA in execution time, while maintaining the same precision. Furthermore, we derived a formula for the DCCA scaling function that is expressed in matrix operations, thus allowing for efficient simultaneous assessment of DCCC from multiple signal pairs. We demonstrated on experimental EEG data that real-time DCCC analysis can be utilized to track mental state of the subjects. Our real-time algorithm may serve as a valuable tool for online neurophysiological data analysis – such as in case of BCI studies –, however its application is not restricted for physiological data but other disciplines as well, where monitoring the dynamics of long-range interactions might be of interest (e.g., financial data analysis).

## Data Availability Statement

The raw data supporting the conclusions of this article will be made available by the authors, without undue reservation.

## Ethics Statement

The studies involving human participants were reviewed and approved by Regional and Institutional Committee of Science and Research Ethics of Semmelweis University. The patients/participants provided their written informed consent to participate in this study.

## Author Contributions

ZK carried out measurements, performed simulations and data analyses, contributed to development of the method, and wrote the first draft of the manuscript. AC, OS, and KK carried out measurements and contributed to data analysis. PM and AE contributed to developing the method and the manuscript. FR conceptualized the study, developed the method, implemented the classification pipeline, and contributed to manuscript writing. All authors have contributed to, reviewed, and approved the final version of the manuscript.

## Conflict of Interest

The authors declare that the research was conducted in the absence of any commercial or financial relationships that could be construed as a potential conflict of interest.

## Publisher’s Note

All claims expressed in this article are solely those of the authors and do not necessarily represent those of their affiliated organizations, or those of the publisher, the editors and the reviewers. Any product that may be evaluated in this article, or claim that may be made by its manufacturer, is not guaranteed or endorsed by the publisher.
